# Supplementary material for: Microbial community structure and carbon transformation characteristics of different aggregates in black soil
Source: PeerJ. 2024 Apr 29;12:e17269. doi: 10.7717/peerj.17269 (PMC11064869; doi:10.7717/peerj.17269)
Supplement: Supplemental Information 1 — Means ± standard errors (n = 3). [file peerj-12-17269-s001.docx]

| **Process** | **Soil grain size** | **SOC（g/kg-1）** | **WSOC（mg/kg-1）** | **HA（g/kg-1）** | **FA（g/kg-1）** |
| --- | --- | --- | --- | --- | --- |
| pre-freeze-thaw | undisturbed soil | 17±1.55 | 53.43±5.34 | 5.20234±0.13 | 4.2636±0.30 |
|  | >5mm | 14.49±3.16 | 81.92±15.65 | 5.31454±0.23 | 3.74±0.23 |
|  | 5-2mm | 8.83±0.99 | 69.46±8.01 | 5.20234±0.13 | 4.4506±0.17 |
|  | 2-1mm | 16.20±1.27 | 60.50±3.65 | 5.56817±0.07 | 4.51812±0.13 |
|  | 1-0.5mm | 11.99±0.91 | 61.44±2.67 | 5.09014±0.28 | 4.3758±0.40 |
|  | 0.5-0.25mm | 17.25±1.04 | 29.92±1.30 | 5.5254±0.20 | 5.07571±0.13 |
|  | <0.25mm | 19.09±0.91 | 22.27±2.02 | 6.22182±0.31 | 4.34195±0.09 |
| post-freeze–thaw | undisturbed soil | 15.10±0.8 | 88.35±7.86 | 5.12±0.13 | 2.51±0.42 |
|  | >5mm | 15.35±2.86 | 80.19±1.46 | 5.32±0.24 | 1.94±0.11 |
|  | 5-2mm | 6.22±0.22 | 87.50±2.57 | 5.12±0.13 | 1.61±0.32 |
|  | 2-1mm | 12.69±2.16 | 79.78±5.15 | 4.90±0.06 | 1.76±0.17 |
|  | 1-0.5mm | 14.21±0.44 | 99.30±1.58 | 4.72±0.27 | 1.66±0.27 |
|  | 0.5-0.25mm | 24.23±1.58 | 49.78±3.21 | 7.31±0.27 | 2.28±0.55 |
|  | <0.25mm | 21.54±2.06 | 76.01±2.45 | 7.43±0.37 | 1.62±0.18 |

**Notes.**

SOC,soil organic carbon;WSOC,water-soluble organic carbon;HA,humic acid;FA,fulvic acid.
